# Assessing the effect of orally administered Ivermectin on viremia and shedding of porcine reproductive and respiratory syndrome virus in growing pigs under field conditions

**DOI:** 10.3389/fvets.2025.1598231

**Published:** 2025-07-22

**Authors:** Grant Allison, Phillip Gauger, Onyekachukwu Osemeke, Paul Lawrence

**Affiliations:** ^1^Walcott Veterinary Clinic, Walcott, IA, United States; ^2^Veterinary Diagnostic Laboratory, Department of Veterinary Diagnostic and Production Animal Medicine, Iowa State University, Ames, IA, United States; ^3^Bimeda Biologicals, Oakdale, MN, United States

**Keywords:** PRRSV, Ivermectin, viremia, shedding, swine, wean-to-market, oral fluids

## Abstract

Despite more than three decades of research and improved biosecurity, porcine reproductive and respiratory syndrome virus (PRRSV) still causes major economic losses to the swine industry. The continued losses, especially in growing-pig populations, prompt exploration of adjunct therapies. This longitudinal field study evaluated whether orally delivered Ivermectin (0.3 mg/kg in four two-day pulses) mitigates PRRSV in a 2,400 wean-to-market barn. Treated pens recorded numerically fewer PRRSV-positive pens during the first 17 days post-weaning, as determined by oral-fluid (OF) RT-qPCR, and lower mean log₁₀ viral copies in OF at eight of nine weekly samplings across 68 days. Serum RT-qPCR, however, revealed numerically higher viremia in treated pigs. These findings warrant further studies using better trial settings or different dosing strategies.

## 1 Introduction

The porcine reproductive and respiratory syndrome virus (PRRSV) remains a global challenge for swine production, resulting in annual losses of hundreds of millions of dollars (1–4). The US swine industry has evolved in many positive ways within the last three decades concerning PRRSV surveillance, diagnostic technologies, prevention strategies, control, and elimination programs (5–9). Despite these advancements, PRRSV continues to undermine herd health and profitability; specific attributes of the virus, such as its ability to sustain low prevalence and its high mutation rates, pose a formidable challenge to traditional control and elimination programs. In the United States, wean-to-market swine herds typically have less rigorous PRRSV surveillance, control, and elimination measures than breeding herds, often making them key reservoirs for the virus and frequent sources of new infections in breeding populations (10–12).

In light of these ongoing challenges, researchers continue to seek novel strategies for mitigating PRRSV’s impact and accelerating its control or elimination in swine populations. One such strategy involves Ivermectin, a commonly used antiparasitic agent in livestock, which has demonstrated ability in inhibiting PRRSV replication in porcine alveolar macrophages *in vitro*, with an ED_50_ of 6.7 μM (13). Similarly, Ivermectin has been shown to inhibit the porcine epidemic diarrhea virus *in vitro* (14, 15).

Ivermectin’s plasma concentration-time profiles following oral administration via feed have been documented (16). One study comparing single- and double-dose nasal spray versus oral administration found Ivermectin in plasma, nasopharyngeal tissue, and lung tissue at 6- and 12-h post-administration, with higher levels detected in lung tissue after oral administration (17). Because the nasal cavity is an entry point for respiratory viruses like PRRSV (18), and early infection can involve both lung and lymphoid tissues (19), interventions that limit PRRSV replication in the lungs may reduce subsequent shedding.

Accordingly, the objective of this study was to assess the effect of orally administering Ivermectin on the downstream reverse transcription-quantitative polymerase chain reaction (RT-qPCR) detection of PRRSV RNA in the serum and oral fluids (OF) of weaned pigs.

## 2 Materials and methods

This longitudinal study was done in the north half of a 2 × 2,400 head tunnel-ventilated wean-to-market barn in the Midwestern USA. Each trial pen (n = 20) was stocked with 115 21-day-old Grade “A” (top) quality pigs farrowed by sows in their second parity or higher and sourced from a batch-farrowing PRRSV-positive unstable sow herd. The piglets were naturally exposed to wild-type PRRSV and were not inoculated for this study. All trial pens were plumbed such that alternating even-numbered pens (n = 10) received Ivermectin in the drinking water, while odd-numbered pens (n = 10) received Ivermectin-free water. Ivermectin treatment in the drinking water began on day 4 post-weaning. Based on the average water intake on day 3 post-weaning, Polysorbate 20 (Food Grade Kosher, FL USA) was mixed with water and Ivermectin to make a stock solution that lasted 24 h.

Ivermectin treatment (0.3 mg/kg/day for 2 days) began on day 4 post-weaning. Based on the prior day’s intake of water and body weight, 3 additional pulses of Ivermectin (on days 11, 21, and 32 post-placement) were given to complete the treatment for the treatment group. Three randomly selected pigs per pen were ear-tagged and subsequently sampled for serum on days 4, 10, 17, 24, 31, 40, and 47 post-placement. OF (obtained by wringing one rope chewed by pigs in the same pen) were collected per pen on the previously described serum sampling schedule but with additional collections on days 54 and 68 post-weaning; during each sampling event, the ropes were tied on the same position on the pen rails for at least 1 h. All samples were sent to a NAHLN-approved veterinary diagnostic laboratory for PRRSV RT-qPCR. Οn day 123 post-weaning, pigs were weighed (30 treatment, 28 control) using a portable electronic scale.

Tables were used to descriptively characterize temporal changes in RT-qPCR detection and log virus concentration of PRRSV in serum and OF. Regression models were used to evaluate statistical differences in the Log_10_ viral concentration and PRRSV RT-qPCR detection rates between the Ivermectin-treated groups and the control groups. These were done using the *Lme4* package (20) in R statistical software (21). In the built models, the Log_10_ viral concentration was used as the outcome variable, the treatment group was a predictor variable (fixed effect), and treatment week was used as a random effect.

## 3 Results

The RFLP pattern and lineage of the circulating PRRSV variant was 1–4-4 L1A. Four days post-weaning, 1.7% (1/60) of the pigs bled were RT-qPCR positive. However, by day 40 post-weaning, 100% (n = 57) of all serum samples were RT-qPCR positive for PRRSV (Table 1).

**Table 1:** Table showing the proportion of PRRSV RT-qPCR positive animals and mean Log_10_ viral concentration per treatment group in serum.

	Percentage of RT-qPCR-positive animals or serum samples (and proportions)		Mean Log_10_ viral copies per ml in serum samples	
Days post-weaning	Control	Ivermectin-treated	Control	Ivermectin-treated
4	3.3% (1/30)	0% (0/30)	0.27	0
10	3.3% (1/30)	3.3% (1/30)	0.25	0.26
17	3.3% (1/30)	10% (3/30)	0.22	0.61
24	36.7% (11/30)	43.3% (13/30)	2.20	2.88
31	96.6% (28/29)	100% (30/30)	6.66	6.69
40	100% (28/28)	100% (29/29)	6.51	6.78
47	53.6% (15/28)	79.3% (23/29)	1.86	2.77

A numerically higher mean Log_10_ viral copies was observed in the control group compared to the treatment groups for 8 out of 9 weeks of oral fluid sampling. We also observed fewer pens shedding PRRSV in oral fluid during the first 17 days post weaning (Table 2). However, there was no statistical difference (*p* > 0.05) between the study groups in Log_10_ viral copies in serum or OF.

**Table 2:** The proportion of PRRSV RT-qPCR positive pens and mean Log_10_ viral concentration per treatment group in oral fluids.

	Percentage of RT-qPCR-positive pens or oral fluids samples (and proportions)		Mean Log_10_ viral copies per ml in positive oral fluids samples	
Days post-weaning	Control	Ivermectin-treated	Control	Ivermectin-treated
4	30% (3/10)	20% (2/10)	0.88	0.80
10	40% (4/10)	30% (3/10)	1.34	1.01
17	80% (8/10)	40% (4/10)	2.63	1.55
24	100% (10/10)	100% (10/10)	4.68	4.48
31	100% (10/10)	100% (10/10)	5.15	5.05
40	100% (10/10)	100% (10/10)	4.67	4.64
47	100% (10/10)	100% (10/10)	4.74	4.75
54	100% (10/10)	100% (10/10)	4.87	4.60
68	100% (10/10)	100% (10/10)	3.97	3.90

Additional findings:

- On day 54 post-weaning, 3 of 3 oral fluid samples collected from both barns were Influenza A virus-positive (Ct 20.4–27.9).
- There was no statistical difference in average weight between treatment (92.4 kg [203.6 lb]) and control (93.9 kg [207 lb]) pigs (*p* = 0.32). Standard deviations differed (treatment 11.2 kg [24.8 lb] vs. control 13.2 kg [29.0 lb]), with 100% of treated pigs and 85.7% of control pigs falling within two standard deviations of their respective means, suggesting greater weight variation in the control group.
- For the first 23 days post-weaning, morbidity or pig treatment (1.2% vs. 1.4%), removals (3.7% vs. 4.3%), and mortality (0.4% vs. 0.3%) were comparable between treatment and control groups, respectively. Average daily gain over the first 123 days post-weaning was likewise similar (0.75 kg [1.66 lb] vs. 0.76 kg [1.68 lb]).

## 4 Discussion

In this field study, numerically higher PRRSV RNA detection in serum was observed in the treated group at 6 of the 7 sampling points, whereas numerically lesser PRRSV RNA was detected in the OF of the treated group at 8 of the 9 sampling points. Fewer pens were shedding PRRSV in OF during the first 17 days post-weaning. It is important to note however that only 3 pigs per pen (out of 115) were sampled for serum, potentially underrepresenting the true dynamics of PRRSV in a relatively large population of pigs. By contrast, OF sampling likely provided a more representative measure of overall PRRSV activity as a significant proportion of pen-mates contribute to the sample.

Our field observations align partially with earlier work. Crawford et al. observed that subcutaneously injected Ivermectin did not have a statistically significant effect on viremia or viral presence in bronchioalveolar lavage but did result in fewer lung lesions (22). Conversely, another study reported a shortened time-to-negative response in serum and OF in mature replacement gilts injected with Ivermectin (23).

Likewise, in the study by Linhares et al., there was no difference in the degree of viremia despite statistically less PRRSV being shed in OF in the vaccine-challenged pigs, indicating that discrepancies in PRRSV RT-qPCR outcomes between serum and OF from the same population of animals are not new (24).

Housing treated and untreated pigs in the same barn may have obscured potential differences. More pronounced effects could have been observed in a study setting where study groups were housed separately.

Based on a previous study (25), a single oral dose of Ivermectin at 0.6 mg/kg achieved a peak plasma concentration (C_max_) of about 20 ng/mL (or 0.0228 μM, assuming the molecular weight of Ivermectin to be 875 g/mol); our field regimen of 0.30 mg/kg/day would therefore have an estimated C_max_ of about 0.011 μM; about 600 folds below the 6.7 μM *in vitro* ED₅₀ for PRRSV reported by Lee et al. (13). The 600-fold comparison is considering a single-day dosing; however, our field regimen involved oral dosing for 8 days [orally administered Ivermectin is reported to have an estimated half-life of about 1.7 days (25)]. Because oral Ivermectin is already licensed, relatively inexpensive, and logistically easy to deliver to large groups of pigs, and anecdotal reports from practitioners suggested a possible reduction in PRRSV shedding even at labelled antiparasitic doses, we considered it scientifically worthwhile to document a carefully monitored “real-world” attempt.

Given the well-documented risk of aerosolized spread of PRRSV, interventions that mitigate the onset and magnitude of PRRSV shedding warrant further investigation, particularly given the inherent biosecurity challenges associated with conventional growing-pig facilities. While our results did not demonstrate a statistically significant effect on viremia or shedding, publishing field observations is valuable considering the economic significance of PRRSV infection to the US swine industry and the absence of consistently effective control strategies. Publishing such reports also highlight logistical considerations, help refine power calculations and dosing strategies for future, higher-concentration or alternative-route studies.

Further research into the use of Ivermectin in the drinking water of weaned pigs is warranted, as it may potentially reduce the shedding of PRRSV, thereby lowering the risk of infection within and between swine populations. Additionally, studies are needed to assess the impact of reduced PRRSV shedding in OF on growth performance. Such investigations may provide further insight to helpful interventions that enhance swine health and productivity while mitigating the economic losses associated with PRRSV infections.

## 5 Conclusion

In this field study, oral administration of Ivermectin to wean-to-market pigs was associated with numerically lower detection of PRRSV in OF at most sampling points, and fewer pens shedding PRRSV in OF during the first 3 weeks post-weaning. In contrast, serum testing showed numerically higher viremia in the treatment group at most time points, although sampling only three pigs per pen may have mis- or underrepresented population-level viremia. No statistically significant differences were detected for either OF or serum. These findings suggest that Ivermectin may help reduce PRRSV shedding; more research is needed to clarify the impact on both viremia and overall viral transmission under commercial conditions.

## Data Availability

The original contributions presented in the study are included in the article/supplementary material, further inquiries can be directed to the corresponding author.
